# Maladie de Still de l’enfant et leucémie aigüe lymphoblastique, une association exceptionnelle: à propos d’un cas

**DOI:** 10.11604/pamj.2022.43.156.33476

**Published:** 2022-11-24

**Authors:** Paul Eloundou, Francine Same Bebey, Ritha Carole Mbono Betoko, Aly Badra Kamissoko, Emeline Tiogouo, Leo Fozeu, Guy Sadeu Wafeu

**Affiliations:** 1 Faculté de Médicine et de Sciences Pharmaceutiques, Université de Douala, Douala, Cameroun,; 2 Centre Hospitalier Universitaire Ignace Deen, Conakry, Guinée Conakry,; 3 Hôpital de District de Biyem Assi, Yaoundé, Cameroun,; 4 Faculté de Médecine et des Sciences Biomédicales, Université de Yaoundé I, Yaoundé, Cameroun,; 5 Centre de Recherche sur les Filarioses et Autres Maladies Tropicales, Yaoundé, Cameroun

**Keywords:** Maladie de Still, enfant, leucémie aigüe lymphoblastique, cas clinique, Still’s disease, child, acute lymphoblastic leukemia, case report

## Abstract

La maladie de Still de l´enfant et la leucémie aigue lymphoblastique ont des similitudes cliniques et biologiques qui posent des problèmes de retard de diagnostic et de prise en charge. En effet, si la maladie de Still est un diagnostic d´exclusion en rhumatologie, la polyarthrite associée à l´hyperleucocytose en contexte fébrile qui la caractérise est souvent rencontrée au cours des leucémies aigues lymphoïdes au stade précoce. Nous rapportons le cas d´une fillette de 4 ans, traitée comme maladie de Still de l´enfant et chez qui le diagnostic de LAL a été posé au bout de 2 mois grâce à une biopsie de la moelle osseuse.

## Abstract (en)

Still’s disease, also known as systemic juvenile idiopathic arthritis (SJIA), and acute lymphoblastic leukemia have similar clinical and biological features posing diagnostic and treatment challenges. Indeed, while Still’s disease is a diagnosis of exclusion in rheumatology, polyarthritis associated with hyperleukocytosis and fever, which is characteristic of this disease, are often detected in early stages of acute lymphocytic leukaemias. We here report the case of a 4-year-old girl, treated for Still’s disease, in whom the diagnosis of acute lymphoblastic leukemia was made after 2 months, based on bone marrow biopsy.

## Introduction

L´arthrite juvénile idiopathique (AJI) est un diagnostic d'exclusion qui englobe toutes les formes d´arthrite débutant avant l'âge de 16 ans, persistant depuis plus de 6 semaines et d´origine inconnue. Ce groupe hétérogène d'arthrites chroniques a été classifié en 2012 par l´ILAR en 06 formes homogènes et mutuellement exclusives grâce aux critères cliniques et biologiques. Il s´agit de la forme systémique ou maladie de Still de l´enfant, la forme poly articulaire avec facteur rhumatoïde négatif, la forme poly articulaire avec facteur rhumatoïde positif, l´arthrite psoriasique, la forme enthésitique et la forme oligo articulaire [1]. La maladie de Still de l´enfant se manifeste cliniquement par une fièvre brutale prolongée, des arthralgies, des arthrites, des dysphagies, un syndrome tumoral fait d´adénopathies et d´hépatosplénomégalie, et rarement d´une hémorragie. Les signes biologiques sont un syndrome inflammatoire très important, une hypertriglycéridémie, une ferritinémie élevée, des LDH élevés, des perturbations de la fonction hépatique en dehors de toute infection documentée [2].

Les signes ostéoarticulaires sus cités peuvent également être retrouvés chez les enfants atteint d´une hémopathie maligne telle que la leucémie aigüe lymphoblastique, affection maligne la plus fréquente chez l´enfant. En effet, 15 à 30 % des enfants souffrant de cancers présentent des troubles musculo squelettiques au moment du diagnostic [3]. Cependant, les diagnostics rhumatologiques sont généralement évoqués de prime abord chez les patients ayant une arthrite chronique, retardant ainsi le diagnostic et le traitement du cancer chez certains patients. De plus, la corticothérapie initiée devant les signes évocateurs de rhumatisme inflammatoire pourrait masquer les signes biologiques de cancer, retardant ainsi le diagnostic et affectant négativement la réponse à une chimiothérapie ultérieure [4]. Nous rapportons un cas de leucémie aigue lymphoblastique chez un enfant traité pour une arthrite juvénile idiopathique dans sa forme systémique ou maladie de Still de l´enfant. Ce cas met en exergue la nécessité d´une approche diagnostic globale incluant la possibilité d´un cancer de l´enfant face aux polyarthrites chroniques.

## Patient et observation

**Information de la patiente:** il s´agit d´un enfant de 4 ans de sexe féminin, troisième enfant d´une fratrie de 5 dont les autres frères étaient en bonne santé apparente.

**Résultats cliniques:** elle a été emmenée en consultation pour une fièvre à 39° C évoluant depuis 15 jours, associée à une léthargie. L´examen clinique ne décelait aucune porte d´entrée infectieuse. L´examen rhumatologique était sans particularité à ce jour.

**Démarche diagnostique:** un syndrome inflammatoire biologique a été retrouvé avec une vitesse de sédimentation à 90 mm/h et la C-Reactive Protein (CRP) à 70 mg/l. L´hémogramme montrait une discrète hyperleucocytose à 11.000 GB/mm^3^ à prédominance neutrophilique à 67% et une anémie microcytaire hypochrome à 8g/dl. Les hémocultures étaient négatives, et l´examen cytobactériologique des urines était stérile. La goutte épaisse était positive avec 700 trophozoïtes de *Plasmodium falciparum* /mm^3^ de sang.

**Intervention thérapeutique et suivi:** elle a reçu un traitement anti paludéen à base d´arthemether par voie parentérale et des antipyrétiques. Au 5^e^ jour de traitement, la température et la CRP était normales, et la goutte épaisse était négative. Sept jours après ce traitement la fillette a été admise en hospitalisation dans un tableau associant une polyarthrite fébrile symétrique non déformante affectant les 2 chevilles, les 2 pieds, les 2 mains et la hanche gauche, un rash cutané morbiliforme, une hépatosplénomégalie modérée. La CRP était à nouveau élevée à 105mg/l, la vitesse de sédimentation à 100mm/h, la ferritinémie à 1475mg/l avec une chute de plus de 20% de sa fraction beta glycosylée. La recherche d´auto anticorps était négative, les CPK étaient normales mais les LDH et les triglycérides étaient élevés. Les fonctions rénales et hépatiques étaient normales. Le frottis sanguin ne montrait pas d´anomalie particulière. Le diagnostic d´arthrite juvénile idiopathique dans sa forme systémique ou maladie de Still de l´enfant a été retenu. La fillette a reçu une corticothérapie et du méthotrexate à 15 mg par semaine.

**Figure 1:**
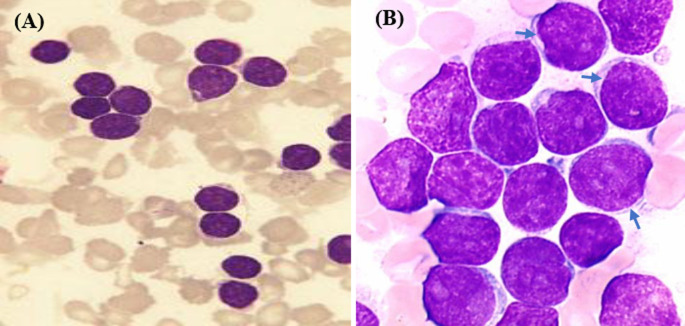
biopsie de la moelle osseuse montrant une prolifération homogène de petits lymphoblastes avec un cytoplasme réduit (flèches): forme dite intermédiaire; (A) image en faible grossissement; (B) image en fort grossissement

Un mois plus tard, l´évolution clinique est marquée par l´installation d´un syndrome d´activation lymphohistiocytaire. En effet, il y avait une persistance de la fièvre, et une hépatosplénomégalie. L´hémogramme retrouvait une anémie persistante à 8,8 g/dl, une thrombopénie à 80 000 plaquettes/mm3 et nombre de globules blanc était augmenté à 15 330 GB/mm3 avec comme formule leucocytaire : 5% de neutrophiles, 50% de lymphocytes, 35% de myéloblastes et 10% de myélocytes. Suite à ces résultats, le méthotrexate et la corticothérapie ont été suspendus et un nouveau frottis sanguin a été réalisé mettant en évidence 40% de blastes jeunes symétriques et des ombres de grumpech. Une biopsie de la moelle osseuse a confirmé le diagnostic de leucémie aiguë lymphoblastique stade 1 intermédiaire (Figure 1). Le protocole fralle B du groupe risque standard été administré. Ce protocole comprend le méthotrexate et la prednisone également utilisé dans la maladie de Still de l´enfant. L´évolution clinique a été favorable et la patiente est actuellement en phase de consolidation.

**Consentement du patient:** nous avons obtenu l´assentiment de la patiente et le consentement parental de sa mère pour la publication de ce cas.

## Discussion

La leucémie aiguë lymphoblastique (LAL) est la néoplasie infantile la plus fréquente. Elle peut présenter des troubles musculosquelettiques sous forme d'arthralgie et d'arthrite, un risque élevé d'erreur et de retard diagnostique [5]. Les manifestations musculo-squelettiques de la LAL comprennent les myalgies, les ostéalgies, les arthralgies et les arthrites. De toutes ces manifestations, l´arthrite et l'arthralgie sont souvent les principaux symptômes rencontrés. Au cours des LAL, l´arthralgie est généralement liée à des lésions périostées des os juxtaarticulaires plutôt que l'infiltration directe des cellules leucémiques dans la synoviale [6]. La LAL est la tumeur maligne la plus fréquemment associé à l'arthrite au stade précoce de la maladie. En effet, 6,4% à 9% des patients avec LAL présentent une arthrite comme signe initial de la maladie [2]. Ninna Brix *et al*. en 2018, se sont intéressés à étudier la particularité de l´arthropathie au cours des LAL. Dans leur série de 237 enfants suivis pour LAL, la prévalence de l'arthrite et de l'arthralgie était similaire. Le siège prépondérant était les hanches les genoux, mais une faible proportion présentait également une atteinte symétrique des mains et des poignets, comme c´était le cas chez notre patiente. Par rapport aux enfants atteints d'arthralgie, les enfants atteints d'arthrite étaient plus souvent mal diagnostiqués et 19 % avaient reçu des injections intra-auriculaires de stéroïdes avant le diagnostic de LAL. Ils avaient également un délai de diagnostic plus long, principalement en raison du retard du premier médecin [6].

Or la démarche diagnostique en rhumatologie, impose d´évoquer chez un patient présentant une mono-arthrite, une arthrite septique comme premier diagnostic à exclure si les symptômes apparaissent soudainement accompagnés de fièvre. Le rhumatisme articulaire aigu, l´AJI, le lupus érythémateux disséminé, et les autres connectivites et vascularites doivent être considérés comme prioritaires chez les patients présentant une polyarthrite. Ainsi, il y a un risque d'erreur de diagnostic avec les rhumatismes inflammatoires en particulier l´AJI, les arthrites septiques et les ostéomyélites. L'AJI est particulièrement intéressante car il s'agit d'un diagnostic d'exclusion, lui-même grevé d´une errance diagnostique, ce qui pourrait augmenter le retard dans l'établissement du diagnostic de LAL chez les patients diagnostiqués à tort avec JIA [7]. L´une des publications les plus anciennes portant sur ce sujet est celle d´A. Bradlow *et al*. publiée en 1991, rapportant trois cas de LLA infantile « déguisée » en AJI, chez qui le délai moyen de la période permettant le diagnostic de LAL était de plus de 4 mois, et dans deux cas, la formule sanguine était normale à la première consultation. En effet, une des manifestations communes à la maladie de Still et à la LAL est bien l´hyperleucocytose [8].

Au cours de la maladie de Still, l´hyperleucocytose à prédominance neutrophile lorsqu´elle est franche, à plus de 15 000/mm^3^ avec polynucléose neutrophile doit faire évoquer le diagnostic lorsque l´enquête infectieuse est négative; elle est présente chez environ 60% des patients [9]. Un article récent suggère que le ratio du nombre de neutrophiles sur le nombre de lymphocytes, au cut-off de 3 serait un très bon moyen de distinguer une infection virale aiguë d´un syndrome de Still, avec une aire sous la courbe de 0,967 [10]. Réciproquement, un ratio inférieur à 3 doit orienter vers un autre diagnostic qu´une maladie de Still; autrement dit, un pourcentage de polynucléaires neutrophiles inférieurs à 60% a de bonnes chances de ne pas être dû à une maladie de Still. La présence d´une myélémie voire même d´une réaction leuco-érythroblastique est possible [8]. Le syndrome d´activation lymphohistiocytaire (SALH), antérieurement appelé syndrome d´activation macrophagique (SAM), est caractérisé par une fièvre, une hépatosplénomégalie, des cytopénies, et la découverte d´images d´hémophagocytose, en particulier dans les organes hématopoïétiques [10]. La physiopathologie du SALH repose sur un défaut de cytotoxicité menant à une activation incontrôlée des macrophages, histiocytes et lymphocytes T et à un « orage cytokinique » au sein duquel l´IL-1 etl´IL-18 ont un rôle déterminant. Par conséquent, plusieurs auteurs suggèrent que le SALH et la MSA systémique partagent le même spectre nosologique, la MSA représentant une forme atténuée.

L'évaluation des frottis sanguins périphériques joue donc un rôle important dans le diagnostic de la LAL. La présence de cellules atypiques dans le sang périphérique peut révéler la présence d'une tumeur maligne, bien que toute anomalie dans la numération globulaire surtout dans la première période ne se voie pas. D´autres parts, l'absence de blastes dans le frottis sanguin périphérique ne doit jamais conduire à l'exclusion des tumeurs malignes. Dans différentes études menées en cet égard, il a été rapporté que les blastes peuvent ne pas être observés dans les frottis sanguins périphériques pour 83% des cas [6]. Chez notre fillette, la biopsie de la moelle osseuse a permis de lever le doute entre une réaction leuco-érythroblastique avec SALH et une authentique leucémie aiguë lymphoblastique.

## Conclusion

Au cours de la maladie de Still de l´enfant, la recherche de blaste dans le sang périphérique peut réduire le délai de diagnostic d´une leucémie aiguë lymphoblastique.

## References

[1] Martini A (2012). It is time to rethink juvenile idiopathic arthritis classification and nomenclature. Ann Rheum Dis.

[2] Prakken B, Albani S, Martini A (2011). Juvenile idiopathic arthritis. Lancet Lond Engl.

[3] Cabral DA, Tucker LB (1999). Malignancies in children who initially present with rheumatic complaints. J Pediatr.

[4] Michel G (2008). Leucémie aiguë lymphoblastique de l´enfant et de l´adolescent: clinique et traitement. EMC_(Elsevier Masson SAS, Paris). Pédiatrie. 4.080.D-10.

[5] Barbosa CMPL, Nakamura C, Terreri MT, Lee ML de M, Petrilli AS, Hilário MOE (2002). [Musculoskeletal manifestations as the onset of acute leukemias in childhood]. J Pediatr (Rio J).

[6] Brix N, Rosthøj S, Herlin T, Hasle H (2015). Arthritis as presenting manifestation of acute lymphoblastic leukaemia in children. Arch Dis Child.

[7] Marwaha RK, Kulkarni KP, Bansal D, Trehan A (2010). Acute lymphoblastic leukemia masquerading as juvenile rheumatoid arthritis: diagnostic pitfall and association with survival. Ann Hematol.

[8] Bradlow A, Barton C (1991). Arthritic presentation of childhood leukaemia. Postgrad Med J.

[9] Mody GM, Cassim B (1997). Rheumatologic manifestations of malignancy. Curr Opin Rheumatol.

[10] Dührsen U, Augener W, Zwingers T, Brittinger G (1987). Spectrum and frequency of autoimmune derangements in lymphoproliferative disorders: analysis of 637 cases and comparison with myeloproliferative diseases. Br J Haematol.

